# RSV Prevention in All Infants: Which Is the Most Preferable Strategy?

**DOI:** 10.3389/fimmu.2022.880368

**Published:** 2022-04-28

**Authors:** Susanna Esposito, Bahaa Abu Raya, Eugenio Baraldi, Katie Flanagan, Federico Martinon Torres, Maria Tsolia, Stefan Zielen

**Affiliations:** ^1^Pediatric Clinic, Pietro Barilla Children’s Hospital, University of Parma, Parma, Italy; ^2^Department of Pediatrics, University of British Columbia, Vancouver, BC, Canada; ^3^Neonatal Intensive Care Unit, Department of Woman’s and Child’s Health, Padova University Hospital, Padova, Italy; ^4^School of Medicine, Faculty of Health Sciences, University of Tasmania, Launceston, TAS, Australia; ^5^School of Health and Biomedical Science, RMIT University, Melbourne, VIC, Australia; ^6^Department of Immunology and Pathology, Monash University, Melbourne, VIC, Australia; ^7^Tasmanian Vaccine Trial Centre, Clifford Craig Foundation, Launceston General Hospital, Launceston, TAS, Australia; ^8^Genetics, Vaccines, Infections and Pediatrics Research group (GENVIP), Hospital Clínico Universitario de Santiago de Compostela, Santiago de Compostela, Spain; ^9^Second Department of Pediatrics, National and Kapodistrian University of Athens, “A&P Kyriakou” Children’s Hospital, Athens, Greece; ^10^Department for Children and Adolescents, Division of Allergology, Pulmonology and Cystic Fibrosis, Goethe-University Hospital, Frankfurt am Main, Germany

**Keywords:** asthma, lower respiratory tract infection, maternal immunization, monoclonal antibody, nirsevimab, palivizumab, prevention, RSV

## Abstract

Respiratory syncytial virus (RSV) causes a spectrum of respiratory illnesses in infants and young children that may lead to hospitalizations and a substantial number of outpatient visits, which result in a huge economic and healthcare burden. Most hospitalizations happen in otherwise healthy infants, highlighting the need to protect all infants against RSV. Moreover, there is evidence on the association between early-life RSV respiratory illness and recurrent wheezing/asthma-like symptoms As such, RSV is considered a global health priority. However, despite this, the only prevention strategy currently available is palivizumab, a monoclonal antibody (mAb) indicated in a subset of preterm infants or those with comorbidities, hence leaving the majority of the infant population unprotected against this virus. Therefore, development of prevention strategies against RSV for all infants entering their first RSV season constitutes a large unmet medical need. The aim of this review is to explore different immunization approaches to protect all infants against RSV. Prevention strategies include maternal immunization, immunization of infants with vaccines, immunization of infants with licensed mAbs (palivizumab), and immunization of infants with long-acting mAbs (e.g., nirsevimab, MK-1654). Of these, palivizumab use is restricted to a small population of infants and does not offer a solution for all-infant protection, whereas vaccine development in infants has encountered various challenges, including the immaturity of the infant immune system, highlighting that future pediatric vaccines will most likely be used in older infants (>6 months of age) and children. Consequently, maternal immunization and immunization of infants with long-acting mAbs represent the two feasible strategies for protection of all infants against RSV. Here, we present considerations regarding these two strategies covering key areas which include mechanism of action, “consistency” of protection, RSV variability, duration of protection, flexibility and optimal timing of immunization, benefit for the mother, programmatic implementation, and acceptance of each strategy by key stakeholders. We conclude that, based on current data, immunization of infants with long-acting mAbs might represent the most effective approach for protecting all infants entering their first RSV season.

## Introduction

Respiratory syncytial virus (RSV) is the most frequent cause of respiratory disease in infants and young children (1, 2). RSV infections are associated with a spectrum of respiratory illnesses, ranging from mild upper respiratory illness to life-threatening bronchiolitis and pneumonia (2). It is estimated that RSV infections account for ~60–80% of infant bronchiolitis and up to 40% of pediatric pneumonias (3). Nearly 70% of infants are infected with RSV in their first year of life, and nearly all children (90%) are infected within the first two years of life, with up to 40% of these developing a lower respiratory tract infection (LRTI) with the initial episode (4). Globally, in 2015, ~12 million episodes of RSV LRTI occurred, resulting in 2.3 million hospitalizations and 43,800 deaths in neonates and infants (<1 year old), demonstrating the significant burden RSV causes in the first year of life (1). Most hospitalizations for RSV occur in otherwise healthy infants born at term (5, 6), representing up to 75% of the hospitalizations due to RSV infections (7–9). Infants are at the highest risk for very severe RSV disease at the time they enter their first RSV season (1, 10, 11). However, there is no way to predict which infants within the otherwise healthy population will develop severe RSV disease and require hospitalization (11). Although hospitalization is an important consequence of RSV illness, RSV is also responsible for a substantial outpatient burden among infants and children, including a considerable number of outpatient and emergency department visits (7, 8). Consequently, RSV disease is associated with a high health and economic burden which has made the development of RSV prevention strategies a major global health priority (12).

Moreover, there is growing evidence to support an association between early-life RSV LRTI and recurrent wheezing/asthma-like symptoms (13, 14). It has been estimated that children with a history of RSV-LRTI have a 2- to 12-fold higher risk of developing pediatric asthma (15). The connection between RSV infection and a developmental trajectory of reduced lung function remains throughout adolescence, suggesting a possible role for RSV in the inception of chronic obstructive pulmonary disease. The mechanisms by which RSV contributes to the onset of wheezing/asthma are not fully understood but appear to relate to injury caused directly by the virus and/or to pre-existing predisposing factors. The prevention of acute RSV infections could improve long-term lung health with reductions in wheezing illness and asthma in the pediatric population (14). In this regard recent interventional studies found a lower risk for wheezing in the first years of age in healthy infants born preterm who were treated with palivizumab (16, 17). The etiological link between RSV infection and the development of asthma has long been debated (18–20), but the question remains whether RSV is a true risk factor or not rather a marker of predisposition to asthma in susceptible individuals. Recent studies assume that RSV infection is more likely to be a trigger of a pre-existing predisposition to asthma (20).

Despite the considerable burden associated with RSV disease, and RSV being considered a global health priority, currently, there is no preventative strategy for all infants and treatment is generally limited to symptomatic relief (21). RSV is also one of the few major causes of severe pediatric infection with no available vaccine (22). Palivizumab, a monoclonal antibody (mAb) licensed for >20 years, is the only prophylaxis available against RSV. However, the indication is restricted to a small subset of the pediatric population comprising those at <35 weeks’ gestational age and up to 6 months of age at the start of the RSV season, and those with certain underlying conditions, leaving the majority of infants unprotected from RSV (23, 24). Depending on guidelines from recommending bodies in individual countries, the group of eligible infants represents ~2% of the birth cohort annually (25). This results in millions of infants remaining at risk of severe or even life-threatening disease every year (1), highlighting the large unmet medical need for preventative strategies for all infants in their first RSV season.

## Which Immunization Strategies Could be Implemented to Protect Infants Entering Their First RSV Season?

### Overview of Future Preventative Strategies for Protection of All Infants Against RSV

Preventative strategies for protection of all infants against RSV that are currently in development include passive immunization of young infants through vaccination of pregnant women (maternal immunization), active immunization of older infants and toddlers, and administration of long acting mAbs to neonates and infants (26).

### Maternal Immunization

Maternal immunization results in the passive transfer of maternal antibodies to the fetus *via* the placenta, a process that increases in efficiency as pregnancy advances (27). Notably, most paediatric vaccines are not administered to infants until ~2 months of age and often require multiple doses to ensure full protection, leaving a critical gap of vulnerability in the first months’ of life (28, 29). Vaccination of pregnant women can offer partial protection for infants against certain pathogens (e.g., influenza, tetanus, diphtheria and pertussis) during this critical gap (29–31). Maternal immunization against influenza protects both pregnant women, who are at increased risk of severe disease and death compared with non-pregnant women (32–34), and their infants until they can be fully protected through vaccination at ~6 months of age (29, 35). Maternal immunization against pertussis is also beneficial for infants as infants receive the first dose of the DTaP (diphtheria, tetanus, acellular pertussis) vaccine from ~2 months followed by another four doses during childhood to ensure protection (36). Importantly, maternal immunization represents the only strategy currently available for protecting neonates and young infants against influenza and pertussis in the first months of life until they are fully vaccinated. Therefore, the World Health Organization recommends that pregnant women are prioritized to receive the seasonal influenza vaccine, and to receive the pertussis vaccine in countries with high infant morbidity or mortality from pertussis (37–39). In addition, the Centre of Disease Control (CDC) recommends COVID-19 vaccination for women who are pregnant (40).

Maternal immunization is a promising potential strategy for protecting infants against RSV (40–42). Recently, a Phase III study in 4,636 pregnant women in their third trimester using a nanoparticle vaccine containing the F protein of RSV (ResVax) demonstrated a favorable safety profile, but did not meet the primary endpoint of reduction in medically significant RSV LRTI through the first 90 days of life in infants (43). However, maternal immunization with RSV F reduced RSV LRTI hospitalizations by 44% through the first 90 days of life. Although the study was not powered to investigate vaccine effectiveness by country, geographic differences were observed with South Africa demonstrating greater disease reduction as compared to the US (43). The reasons this maternal vaccine did not meet the prespecified success criterion to provide consistent efficacy against RSV are not fully understood. Further reasons for the apparent lower efficacy in high-income countries might include hospitalization for less severe cases, lower prevalence of breast-feeding, and lower background rates of severe RSV-associated LRTI because of factors such as less exposure to indoor smoke or to crowding and later introduction to social contact (43). However, antibody levels and function that correlate with protection may not have been achieved (44).

Following the RSV F Phase III study in pregnant women, a number of RSV maternal vaccines are currently in development. The pre-fusion conformation of RSV protein (pre-F) is being investigated as an antigen in maternal vaccine formulations as it has the potential to elicit high RSV neutralizing antibody titers, which have been found to correlate with reduced disease severity (26). Specifically, the RSVPreF3 vaccine (GSK) is currently being investigated in a Phase III study (GRACE) (45) to evaluate its effect on medically assessed RSV-associated LRTIs in infants up to 6 months of age. RSVPreF3 is well tolerated and immunogenic in non-pregnant women (46), and has also been shown to increase maternal RSV-specific antibody responses and RSV-A/RSV-B neutralizing antibody titres in mothers and infants (47). Another candidate for maternal immunization against RSV is the RSVPreF vaccine (Pfizer) which is currently being investigated in a Phase III study for its protective effect against RSV in infants up to 6 months (48). Thus far, it has been shown that RSVPreF is well-tolerated and produces robust neutralizing antibody responses in pregnant women with efficient transplacental transfer (49).

### Immunization of Infants

Currently, there is no available vaccine for RSV, and RSV vaccine development for infants has been particularly challenging for several reasons (26). Infants under 4–6 months may have an impaired ability to generate effective, long-lived adaptive memory responses following immunization (26) In line with this, it has been shown that natural RSV infection produces a low immune response in young children <18 months old (50). A second challenge for RSV vaccine development in infants is the safety concern regarding the potential for vaccine enhanced respiratory disease (ERD) when vaccinated children are infected with RSV (26). In the 1960s, the clinical trial failure of a formalin-inactivated RSV vaccine in infants (2–7 month old) who developed ERD following natural RSV infection highlighted this concern (51–54). This is relevant for the naïve infant population, but not the older RSV seropositive children (55). Lastly, a very narrow time frame between birth and first RSV infection has also been a barrier for direct RSV vaccination in infants (8, 26). Due to these challenges, a pediatric vaccine would not be able to fulfil the current unmet need for protecting all infants against RSV from birth.

At present, a variety of vaccine strategies for protection against RSV in infants are being investigated. Vaccines under development include protein vaccines that use stabilized pre-F protein subunits or virus-like particles, and live vaccines that include attenuated RSV strains, or virus vectors expressing RSV proteins (55). The use of each vaccine type will depend on age and also whether children are naïve (or have previous exposure to RSV). Currently, there are 10 pediatric vaccine candidates in clinical development (preclinical, Phase I and II), with the vaccines in Phase II targeting infants >6 months of age (47). Recently, development of a pediatric vaccine, GSK3389245A, also known as ChAd155-RSV, was discontinued at the Phase I/II stage due to lack of expected immunogenicity against RSV (56). In the future, vaccines could serve as an immunization strategy against RSV for older infants/children in the second or third RSV season providing durable protection against RSV throughout childhood (57).

### Immunization of Infants With Licenced mAbs

Palivizumab is the only mAb approved for prophylactic use against RSV, although it is restricted for use in a small group of infants (24, 58). Several studies have demonstrated that RSV hospitalization rates decrease significantly in extreme preterm neonates and infants with comorbidities who are at high risk for RSV. A previous study used representative US hospital data to examine trends in RSV hospitalization in pre-term infants with chronic lung disease (CLD) and found a 48% reduction in RSV hospitalization between 1998 and 2008 (59). Other studies with a more robust database, additional comparison groups, and longer follow-up confirm these results (60–62). A significant reduction in RSV bronchiolitis was found not only in pre-term infants with CLD, but also in children with severe coronary artery disease (60). Interestingly, there was a significant effect of palivizumab on late effects of RSV bronchiolitis. Several well-controlled, randomized, double-blind studies on the prevention of RSV bronchiolitis have shown that palivizumab protects against recurrent obstructive episodes and, over the long term, against the development of childhood asthma (61, 62).

Palivizumab is a recombinant, humanized mAb targeting the fusion (F) protein of the virus by recognizing an epitope within antigenic site II, which is preserved on both the prefusion and post-fusion F conformations of the F protein (63). It has a half-life of 19–27 days, and so requires monthly injections to maintain protection over the RSV season (64). Adherence to the monthly dosing schedule is necessary for maintaining protection against RSV, and although there is no standardized definition of adherence to palivizumab (65), low adherence has been recorded in certain populations (e.g., minority groups) (66).

### Immunization of Infants With Long-Acting mAbs – Under Development

Palivizumab provided the proof of concept that mAbs can be used to prevent RSV disease, and there are ongoing efforts to produce mAbs that provide a more sustained protective effect against RSV than licenced mAbs (67, 68). As such, several long-acting mAbs are in clinical development for RSV prophylaxis in infants, including at least four mAbs in early development and two in late-stage development (nirsevimab and MK-1654) (56). Nirsevimab is a recombinant, human mAb that contains a three amino acid YTE (M252Y/S254T/T256E) substitution in the Fc region, which increases IgG affinity for the human FcRn at lower pH and allows for recirculation of the mAb, resulting in an extended half-life of 63–73 days in infants (69, 70). It is characterized by high potency, neutralizing both RSV-A and RSV-B strains with >50-fold higher affinity compared with palivizumab (70). Nirsevimab’s mechanism of action allows for rapid and direct protection with a single intramuscular dose throughout the RSV season (69, 70). In a Phase IIb study, nirsevimab reduced medically attended RSV-associated LRTI by ~70% and RSV hospitalizations by ~78% versus placebo in healthy preterm infants (71). These results were consistent for the duration of 150 days, which covers the length of a typical RSV season, following administration of a single nirsevimab dose, as well as across different geographic locations and RSV subtypes (RSV-A and RSV-B). Adverse events (AEs) (incidence and types) were similar between nirsevimab and placebo, and antidrug antibodies (ADA) were observed in 5.6% of infants receiving nirsevimab versus 3.8% of those receiving placebo with no differences between groups when AEs were analyzed based on ADA (positive or negative) status (71). In a recent Phase III study (MELODY), nirsevimab reduced medically attended RSV-associated LRTI compared with placebo in healthy preterm and term infants (efficacy 74.5%, p <0.0001) and demonstrated a favorable safety profile (72, 73). A Phase II/III study (MEDLEY) demonstrated a similar safety and tolerability profile compared with palivizumab in preterm infants or those with chronic lung disease or CHD in their first RSV season (74). Based on positive clinical data, nirsevimab could be used to protect against RSV for all infants. In line with this, mathematical modelling has demonstrated that a single dose of nirsevimab administered to all infants entering their first RSV season has the potential to provide significant protection against RSV (75).

Another long-acting mAb currently in development for protection against RSV in infants is MK-1654, which is a fully human mAb targeting site IV of the F protein (76). In a study in healthy adults, MK-1654 serum concentrations increased with dose and resulted in increased RSV serum-neutralizing antibody titers (77). The antibody’s half-life was 73–88 days with an estimated bioavailability of 69% at the dose of 300 mg, and the safety profile of MK-1654 was similar to placebo (77). MK-1654 is currently in a Phase IIb/III study to evaluate efficacy and safety in healthy preterm and term infants (78).

### Future Preventative Strategies for Protection of All Infants Against RSV

**Figure 1:**
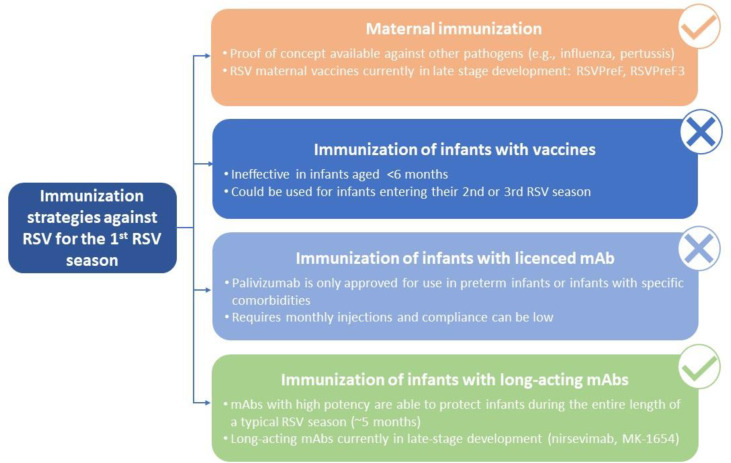
Potential immunization strategies against RSV for infants entering their first RSV season.

Taken together, although immunization with vaccines represents a promising strategy for RSV prophylaxis in infants >6 months of age, clinical and immunological data have demonstrated that this option would most likely not be feasible in neonates and young infants entering their first RSV season. Immunization with licensed mAbs (i.e., palivizumab) requires monthly injections and is indicated for a highly restricted population of infants at risk, which means that the majority of infants would remain unprotected from RSV. Therefore, protection of all infants before they reach 6 months of age (i.e., from birth and potentially covering the entire first year of life) can only be achieved by maternal immunization or immunization with long-acting mAbs (**Figure 1**), which are the focus of the next part of this review.

## Considerations of the Different Strategies for Protecting All Infants Against RSV

### Antibody Transport to the Lumen of the Infant Respiratory Tract

Maternal antibodies produced as a result of active immunization of the mother during pregnancy are transferred to the fetus *via* the placenta (79). The transfer of antibodies is an active process involving the binding of the IgG Fc to the neonatal Fc receptor in the placenta syncytiotrophoblast, but the mechanisms used to transport IgG (predominantly IgG1 isotype) through the final placental layers are not yet fully known. Maternal IgG is transferred across the placenta from approximately 13 weeks’ gestation with the concentration of IgG that is transferred increases sharply during the third trimester and peaks in the final four weeks before birth (79). Fetus/infant antibody titers appear ~2 weeks following maternal vaccination (27). By contrast, mAbs are delivered by intramuscular injection and rapidly reach the infant bloodstream (64, 69, 71, 77).

To prevent RSV infection, IgG antibodies, both maternal or monoclonal, need to reach the respiratory tract lumen, where RSV is encountered. Epithelial cells lining the respiratory tract and other mucosal surfaces, as well as the placenta, are polarized, with apical and basolateral plasma membrane domains, separated by intercellular tight junctions. The neonatal Fc receptor is expressed by mucosal epithelial cells and mediates the transfer (transcytosis) of IgG antibodies (maternal or monoclonal IgG) across the epithelium (80–82). However, systemically administered IgG levels in bronchoalveolar lavage fluid have been reported to be significantly lower than corresponding levels of IgG in serum (83, 84).

### Correlates of Protection

RSV neutralizing antibody levels in cord blood have been associated with protection from RSV hospitalization among infants aged <6 months (42, 85–87), suggesting that neutralization potentially correlates with protection. The antibodies produced as a result of active immunization target multiple epitopes (85) potentially leading to broad coverage; however, it is likely that maternal antibody responses are only partially protective with only a fraction displaying RSV-neutralizing activity (86). Immunization of infants with long-acting mAbs is based on the use of human neutralizing antibodies engineered to display a longer half-life with respect to other mAbs, and prevent RSV entry into human host cells (87). Both nirsevimab and MK-1654 are highly potent neutralizing mAbs against the F protein of the virus, targeting the antigenic site Ø and IV, respectively (70, 76). Nirsevimab, for instance, neutralizes viral entry into host cells by binding to the prefusion form of F protein and inhibiting its conformational change to the post-fusion form (88).

**Figure 2:**
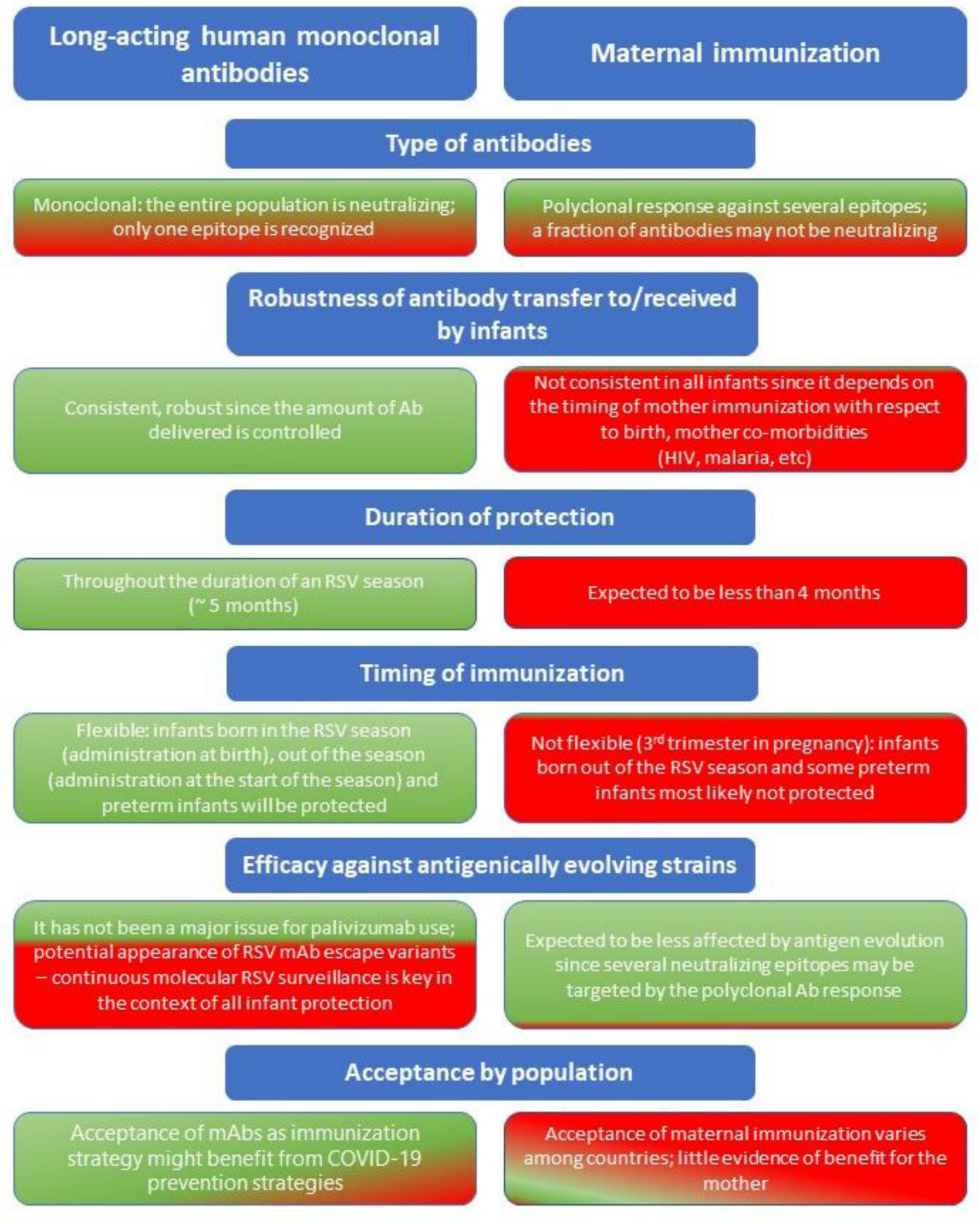
Considerations regarding long-acting human monoclonal antibodies and maternal immunization as prevention strategies against RSV for all infants entering their first RSV season. Green = advantage; Red = disadvantage.

Apart from virus neutralization, other potential functions that may correlate with protection include Fc-mediated antibody effector functions e.g., antibody-dependent cell-mediated cytotoxicity, antibody-dependent cellular phagocytosis, and complement-dependent cytotoxicity (89). However, these mechanisms have not yet been demonstrated for anti-RSV maternal or engineered monoclonal antibodies. MK-1654, for instance, is thought to be efficient only through its neutralizing activity since deletion of its Fc region did not significantly modify its efficacy in a preclinical model (76). Moreover, some of the antibodies after maternal immunization may not be neutralizing, but they may have other Fc-mediated functions. Antibodies that target the G protein of the virus and inhibit viral attachment, as well as mixtures of antibodies against F and G or against two different antigenic sites on F are also in development (**Figure 2**) (88–91).

### Inhibition of RSV Transmission

Based on modelling of RSV transmission in a low-income country, vaccination of pregnant women and household co-habitants against RSV can reduce infant hospitalizations (92). However, there is little evidence to suggest that maternal immunization alone can offer indirect protection to the infant by reducing transmission. Indeed, RSV carried by older siblings or other children in the community i.e. day-care exposure are important sources of infection in infants (93, 94). Furthermore, based on modelling capturing RSV transmission in high-income settings, the impact of maternal immunization reduces as household size increases (95). Consequently, based on current data, it is not likely that protection of the mother against RSV through maternal immunization alone can translate to protection of the infant in an environment where other important sources of infection exist.

### Predicted Protection With Different Strategies

For maternal immunization, a variety of factors, both intrinsic and extrinsic, influence the production of sufficient maternal antibody quantities, and also efficient transplacental transfer (79). IgG subclass, antigen specificity and glycosylation status affect maternal antibody transfer across the placenta; IgG1 is transferred more efficiently than IgG2, IgG3 and IgG4, protein-vaccine-specific antibodies are transferred more efficiently than polysaccharide-vaccine-specific antibodies, and different IgG glycosylated variants show different kinetics and binding affinity to placental Fc receptors (96, 97). As maternal antibody transfer occurs at a minimal degree in the first trimester and shows the highest rates in the last 4 weeks of pregnancy, efficiency of maternal immunization depends on the timing of immunisation prior to delivery (79). Therefore, some preterm infants might not receive adequate maternal antibody titers to be protected against RSV if the mother is not immunized enough in pregnancy (98). This concern is supported by evidence with other vaccine antigens, which shows that preterm infants have lower placental transfer ratios of antibodies against pertussis, diphtheria, tetanus, *Haemophilus influenzae* type b and *Neisseria meningitidis* versus term infants (97). Timing of maternal immunization during pregnancy can also affect the avidity of maternal antibodies produced, as shown for pertussis, with maternal vaccination 5–12 weeks before delivery associated with higher IgG avidity versus vaccination within 4 weeks of delivery (99). In addition, it has been shown that infants with low birthweight, even those born at term, have reduced maternal antibodies, which could be due to placental pathologies often linked to premature birth and intrauterine growth retardation (79, 100, 101). Furthermore, multiple pregnancies are more likely to result in preterm birth than singleton pregnancies (102), which could affect maternal-fetal antibody transfer. Chronic maternal infections such as human immunodeficiency virus (HIV) infection and malaria, and conditions such as hypergammaglobulinemia and primary immunodeficiencies can also impair antibody production, and transfer of maternal IgG (79, 103–108). For example, it has been shown that maternal HIV infection results in lower placental transfer of RSV neutralizing antibodies and lower titers of RSV antibodies in HIV-exposed, uninfected neonates compared with unexposed, uninfected neonates (109).

In contrast, immunization using mAbs is not affected by factors associated with the mother’s health, transplacental transfer of maternal antibodies or placental integrity, as it involves direct administration of mAbs to infants providing more predictable kinetics in infants (**Figure 2**) (70, 71, 110).

### Possible Modulation of Subsequent Active Immunization

The presence of maternal antibodies in the infant have been shown to modulate their antibody response after immunization with certain vaccines, which may result in lower vaccine-induced antibody levels (79). This has been shown for tetanus, pertussis, measles, mumps, diphtheria and influenza immunization (111, 112). Mechanisms of inhibition of immune response by passively-transferred IgG antibodies include inhibition of B cell responses, removal of vaccine antigen by macrophages and neutralization of viruses (98, 112–115). However, maternal antibody levels reduce over time as antibodies are metabolized, therefore, this interference is transient (98, 112). In the case of long-acting mAbs, there is limited published evidence on potential inhibition of the immune response by mAbs. The question of whether modification of primary immunization against RSV applies to mAbs or maternal immunization will be relevant when RSV vaccines become available for use in infants. Immunogenicity and long-term studies are required, including RSV surveillance data collected where maternal immunization or mAbs have been received.

### RSV Genetic Variability

RSV has two major subtypes, A and B, mainly based on differences in the glycoprotein G sequence, and multiple genotypes that can co-circulate during the RSV season (115). RSV is continuously evolving leading to the emergence of new genotypes and the disappearance of older ones (116). Genetic modifications have been detected mostly in the RSV G gene, whereas the F glycoprotein sequence is highly conserved and therefore is used as a target for many anti-RSV mAbs in development (116). However, amino acid changes in neutralizing antigenic sites in the F protein of RSV have been detected (117), raising the concern that mutations may result in variants escaping mAb prophylaxis (118). In line with this, the development of suptavumab for RSV prophylaxis in preterm infants was discontinued due to its lack of efficacy in a Phase III study, caused by escape mutants with amino acid changes in the suptavumab epitope in all circulating RSV B strains (119). Polymorphisms in the nirsevimab and palivizumab binding regions have been reported, and although some of these have not been evaluated in neutralization assays, the frequency of isolates with amino acid changes leading to partial resistance is low (118). Specifically, in a recent analysis of within-host RSV diversity (2017–2020), only 0.8% (2/264) of immunoprophylaxis-naïve participants had RSV-B sequences containing the amino acid change K68N in the F protein, which has been linked to reduced susceptibility to nirsevimab (120). Despite the potential of neutralization escape mutants, clinical use of palivizumab demonstrated that this has not been a major issue (2). Initial analysis of 371 RSV isolates revealed conservation of its neutralizing epitope of the F protein (121), while other studies identified RSV escape mutants in ~5% of 146 breakthrough cases (122), suggesting that escape variants are still uncommon and selective pressure is weak. Therefore, mAbs can show sustained efficacy during long-term clinical use, provided they are specific for a stable epitope of RSV (nirsevimab binding epitopes: amino acids 62–69, 196–212). Nevertheless, ongoing molecular surveillance of RSV globally is key, with current initiatives including the WHO Global RSV Surveillance program, the OUTSMART, and INFORM programs (**Figure 2**) (123–125).

### Duration of Protection Against RSV, Flexibility of Immunization and Optimal Timing of Immunization

**Figure 3:**
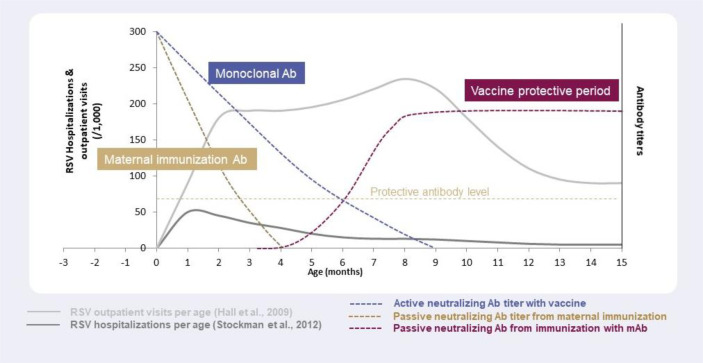
Antibody levels and duration of protection against RSV with different strategies: maternal immunization, immunization with monoclonal antibodies, and immunization with vaccines, in relation to RSV outpatient visits and hospitalizations per age.

Maternal antibodies that are transferred to infants through the placenta may last 2–4 months (126), with the maximum concentration of maternal antibodies present at birth and decay kinetics being key determinants of how long these antibodies can provide protection in infants (127, 128). Since a typical RSV season may last ~5 months (129), protection against RSV *via* maternal antibodies might only cover the first few weeks of life, potentially leaving infants vulnerable to RSV disease for a large part of the season (**Figure 3**). In line with this, the estimated antibody half-life in infants born to mothers vaccinated with an RSV F vaccine candidate, which failed in its Phase III study, was 49.1 days and 38.3 days for palivizumab-competitive antibodies and anti-F IgG, respectively (43). Durability of protection in the infant with new maternal vaccines in development (RSVPreF3, RSVPreF) needs to be defined. Recently, it was shown that RSV-A and RSV-B antibody titers were higher in infants whose mothers had received RSVPreF3 vaccination versus placebo throughout 6 months (47), but no correlation was demonstrated between these antibody levels and clinical impact. Mathematical modelling has demonstrated limited duration and impact of maternally acquired passive immunity for RSV, with maternal immunization estimated to reduce RSV hospitalizations by 6–37% for infants aged 0–2 months, and 30–46% for infants aged 3–5 months, taking into account different vaccine effectiveness levels (130). Regarding flexibility of maternal immunization, although duration of protection could be longer with breastfeeding, based on the duration of maternal antibodies (most likely 2–4 months) only infants born just before and during the RSV season will be protected against RSV, whereas infants born out of the RSV season will not maintain sufficient protection for the entire period of risk (**Figure 2**) (57).

In the case of mAbs administered to infants, nirsevimab’s duration of protection is at least 5 months (97), enabling coverage of the entire RSV season by providing direct and rapid protection for all infants for the whole period of risk (**Figure 3**) (69, 70, 129, 130). In addition, it offers a high degree of flexibility regarding the timing of immunization in terms of RSV seasonality i.e., it can be administered to infants throughout the year as needed depending on the RSV seasonal pattern (57, 107). For infants born outside the RSV season, nirsevimab can be administered just before the start of the season, whereas infants born during the RSV season can be immunized at birth (57, 107). Recently, the impact of COVID-19 on RSV seasonality has highlighted the need for flexibility in terms of immunizing and protecting infants against RSV. Non-pharmaceutical interventions implemented during the COVID-19 pandemic disrupted seasonal patterns of RSV (and other respiratory viruses) by causing a seasonal shift and large interseasonal outbreaks of RSV when measures, such as social distancing, were lifted (131, 132). In light of this, immunization of infants through long-acting mAbs have the advantage of protecting against RSV over the whole infancy period (up to 12 months of age) (**Figure 2**).

### Benefit for the Mother

Thus far, there are limited data available in regards to the incidence of RSV infection in pregnant women, and although RSV can cause symptomatic disease in pregnant women (133), it is frequently mild (133). Notably, RSV infection has only been detected in a small percentage of pregnant women who present with respiratory illness (134–136); however, it can occasionally be detected in women with acute respiratory failure (133). Based on current data, benefits for pregnant women from maternal immunisation against RSV are not clear. In addition, as available data are based on influenza studies, and RSV and influenza seasons do not show complete overlap, further RSV-targeted studies are needed to investigate RSV burden during pregnancy (**Figure 2**) (133, 137).

### Programmatic Implementation

Implementation strategies for maternal immunization and long-acting mAbs depend on the healthcare setting, country-specific guidelines and RSV seasonal patterns (107, 138). For infants expected to be born just before or during the RSV season, a maternal RSV strategy could follow a similar approach as for maternal immunization against influenza and pertussis (48). In theory, as existing settings are already in use, planning for maternal immunization could be straightforward, although in countries and communities with poor maternal vaccine coverage, implementation could be challenging (122). Conversely, infants born a few months before the RSV season would not be protected by maternal immunization due to the decrease of RSV-specific maternal antibody titres, which have a shorter half-life than the mAbs in development. For in-season born infants, administration of long-acting mAbs could take place immediately after birth or during the pre-discharge visit of newborns (70, 71). For out-of-season births, administration could take place at routine pediatric immunization visits or parents could be contacted for immunization of infants before the start of the RSV season (71). Since the use of mAbs is a novel approach for immunization of infants against RSV, all relevant stakeholders (HCPs, care givers, policy makers) would need to be informed of the benefits and need for mAbs in RSV protection, and contribute to the different implementation strategies. Nevertheless, implementation could be straightforward if existing pathways for routine pediatric immunization are used (122).

### Acceptance of Maternal Immunization and Immunization of Infants With Long-Acting mAbs

Considerable variability among countries has been documented in terms of vaccine coverage for influenza and pertussis during pregnancy (139). For maternal influenza vaccination, studies have shown uptake rates of 78% and 76%, in the US and New Zealand, respectively (140), whereas significantly lower coverage has been reported in Greece (16.2%) and Italy (6.5%) (141, 142). For pertussis maternal vaccination, uptake rates range from 74%, 63% and 64% in Taiwan, Australia and Belgium, respectively, to 0% in Greece (141, 143–145). It has been shown that recommendation of maternal vaccination by healthcare professionals (HCPs) is a main facilitator of vaccine coverage during pregnancy (139), with safety concerns among pregnant women and HCPs being common barriers to maternal vaccine uptake (141). Therefore, based on previous experience with maternal vaccines, it is likely that consistent, high maternal vaccine coverage for RSV will be a challenge, which could compromise prevention of RSV in all infants. Immunization of all infants through long-acting mAbs represents a novel strategy, and as such could also encounter challenges regarding acceptance by parents (146–148). However, it is likely that research into COVID-19 prevention strategies will help increase and accelerate acceptance of the use of mAbs as prophylaxis for RSV (122), necessitating education of key stakeholders, including, where applicable, leveraging the messaging around COVID-19 and the increased awareness of the need for immunization currently held by the wider population (**Figure 2**).

## Conclusions

Use of vaccines to protect neonates and young infants against RSV has many challenges, and the only licenced prophylactic mAb available, palivizumab, requires monthly injections and is restricted to a very limited subset of infants. It is unclear whether a maternal immunization strategy would provide only indirect protection of the infant or a direct benefit for the mother. Although maternal vaccination has been successful in some countries and implementation of this strategy against RSV could be based on settings/infrastructure already in place for other vaccines (e.g., influenza, pertussis), hence not requiring substantial planning, previous experience has demonstrated that acceptance of maternal immunization among pregnant women could be a barrier for successful implementation. Immunization of neonates and infants with long-acting mAbs provides consistent, direct protection for at least 5 months against RSV without the requirement for mature immunity from infants, covering the duration of a typical RSV season. It offers a great degree of flexibility regarding timing of administration, which means that infants born out of the RSV season could still be protected through a single injection using a vaccine-like approach. Implementation of long-acting mAbs as an immunization strategy against RSV could be straightforward if existing infrastructure for routine pediatric immunization is used, not requiring additional healthcare visits. Although there needs to be surveillance of RSV variability in case mutations affect immunization targets for mAbs, long-acting mAbs represent the most effective strategy for protecting all infants entering their first RSV season. Future immunization with vaccines might eventually be in place for children >6 months of age to protect toddlers entering their second/third RSV season, providing longer term protection in older children. Taken together, the huge health and economic burden of RSV has made the development and implementation of protection strategies for RSV a high global health priority, with an urgency to protect all infants. The most effective option for protecting all infants against RSV might be the use of long-acting mAbs, highlighting the need to adopt this new technology. Ideally, national/country guidelines should be updated to reflect this, and all key stakeholders (parents, HCPs, policy makers) should be informed of the benefits of these technologies to ensure successful implementation in future immunization programs.

## Author Contributions

SE proposed the project, coordinated the study group and wrote the first draft of the manuscript. BAR, EB, FT KF, MT, and SZ reviewed and edited the manuscript, provided comments and suggested references and substantially contributed to the content of the manuscript. All the authors approved the final version of the manuscript.

## Funding

The publication of this manuscript was supported by the World Association for Infectious Diseases and Immunological Disorders (WAidid).

## Conflict of Interest

SE: Speaker’s fees from GSK, Pfizer, Novartis, Sanofi Pasteur, MSD and Vifor in the past three years. BA is supported by the Michael Smith Foundation for Health Research and received payment as a scientific expert of the Sanofi group of companies. KF is a member of the Australian Technical Advisory Group on Immunisation noting that this paper represents her own personal view; received honoraria as a member of the vaccine advisory boards for Seqiris and Sanofi Pasteur in the last 5 years.

The remaining authors declare that the research was conducted in the absence of any commercial or financial relationships that could be construed as a potential conflict of interest.

## Publisher’s Note

All claims expressed in this article are solely those of the authors and do not necessarily represent those of their affiliated organizations, or those of the publisher, the editors and the reviewers. Any product that may be evaluated in this article, or claim that may be made by its manufacturer, is not guaranteed or endorsed by the publisher.
